# Evaluating the effect of emergency department crowding on triage destination

**DOI:** 10.1186/1865-1380-7-16

**Published:** 2014-04-28

**Authors:** Erin O’Connor, Mathieu Gatien, Cindy Weir, Lisa Calder

**Affiliations:** 1 Department of Emergency Medicine, University of Ottawa, 1053 Carling Avenue, Ottawa, ON K1Y 4E9, Canada; 2 Clinical Epidemiology Program, Ottawa Hospital Research Institute, The Ottawa Hospital, Civic Campus, Rm F658, 1053 Carling Ave., Ottawa, ON K1Y 4E9, Canada

**Keywords:** Emergency Department crowding, Emergency Department occupancy, High acuity, Overcrowding, Triage

## Abstract

**Background:**

Emergency Department (ED) crowding has been studied for the last 20 years, yet many questions remain about its impact on patient care. In this study, we aimed to determine if ED crowding influenced patient triage destination and intensity of investigation, as well as rates of unscheduled returns to the ED. We focused on patients presenting with chest pain or shortness of breath, triaged as high acuity, and who were subsequently discharged home.

**Methods:**

This pilot study was a health records review of 500 patients presenting to two urban tertiary care EDs with chest pain or shortness of breath, triaged as high acuity and subsequently discharged home. Data extracted included triage time, date, treatment area, time to physician initial assessment, investigations ordered, disposition, and return ED visits within 14 days. We defined ED crowding as ED occupancy greater than 1.5. Data were analyzed using descriptive statistics and the χ^2^ and Fisher exact tests.

**Results:**

Over half of the patients, 260/500 (52.0%) presented during conditions of ED crowding. More patients were triaged to the non-monitored area of the ED during ED crowding (65/260 (25.0%) vs. 39/240 (16.3%) when not crowded, *P* = 0.02). During ED crowding, mean time to physician initial assessment was 132.0 minutes in the non-monitored area vs. 99.1 minutes in the monitored area, *P* <0.0001. When the ED was not crowded, mean time to physician initial assessment was 122.3 minutes in the non-monitored area vs. 67 minutes in the monitored area, *P* = 0.0003. Patients did not return to the ED more often when triaged during ED crowding: 24/260 (9.3%) vs. 29/240 (12.1%) when ED was not crowded (*P* = 0.31). Overall, when triaged to the non-monitored area of the ED, 44/396 (11.1%) patients returned, whereas in the monitored area 9/104 (8.7%) patients returned, *P* = 0.46.

**Conclusions:**

ED crowding conditions appeared to influence triage destination in our ED leading to longer wait times for high acuity patients. This did not appear to lead to higher rates of return ED visits amongst discharged patients in this cohort. Further research is needed to determine whether these delays lead to adverse patient outcomes.

## Background

Emergency Department (ED) crowding was first identified as a problem over 20 years ago [1]. Research has since focused on the effect of ED crowding on adverse patient outcomes; however, the effect of ED crowding on triage destination has not been studied. Triage and the assignment of the patient to an area of the ED is an important part of a patient’s visit to an ED. Triage destination can greatly influence the course of the patient’s visit, including time to assessment, extent of workup, and length of stay in the ED [2]. Assignment of a triage score, and subsequent placement in an non-monitored (less acute) vs. monitored (more acute) area of the ED affects physician thinking and decision making about the patient’s presentation [3,4].

ED crowding is a concern with regard to patient safety, as it has been associated with adverse patient outcomes including increased patient mortality, delayed resuscitation efforts, increased adverse events, delayed antibiotic administration in patients with pneumonia, and increased in-hospital length of stay [5-10]. ED crowding has also been related to poor management of pain and decrease in patient satisfaction [11-13].

We present a pilot study of patients presenting with chest pain or shortness of breath, triaged as high acuity, and who were discharged home on the index visit. Our objectives were to determine whether: 1) patients were triaged to non-monitored areas of the ED more frequently during ED crowding; 2) patients were assessed by a physician more quickly in non-monitored rather than monitored areas during crowded conditions; 3) patients triaged to the non-monitored area received the same laboratory and imaging tests as those triaged to monitored areas; 4) patients triaged during ED crowding received the same laboratory and imaging tests as those triaged during non-crowded conditions; 5) the proportion of return ED visits was higher for patients triaged during ED crowding; and 6) the proportion of return ED visits was higher for patients triaged to the non-monitored area during crowded conditions.

## Methods

### Study design and setting

We conducted a health records review of patients presenting to two ED campuses of a large urban tertiary care Canadian academic teaching hospital, each with approximately 75,000 patient visits per year. We used International Classification of Diseases 10 codes to identify patient visits corresponding with chief complaints of chest pain or shortness of breath for the period between January 1^st^ and December 31^st^ 2010. A total number of 4,234 patient visits were identified. Using an Internet-based random number generator (http://www.randomizer.org), we selected a sample of health records, with an overall goal of 500 eligible visits. The Ottawa Hospital Research Ethics Board approved this study.

### Subjects and data collection

We included patients older than 18, who presented with a chief complaint of either chest pain or shortness of breath, were assigned a Canadian Triage and Acuity Scale (CTAS) score of 2, and were discharged home on the index visit [14]. CTAS is a five-category triage system designed to allow Canadian EDs to prioritize patients based on their presenting complaint and the severity of their signs and symptoms. Triage nurses who have received training assign CTAS scores based on a published set of guidelines [14]. CTAS scores are determined by presenting complaint, with severity modifiers based on vital signs, past medical history, and brief history of the complaint [15]. Included in the CTAS score assignment system is nursing discretion, allowing for a higher CTAS score to be assigned based on the judgment of the triage nurse. In our hospital there are guidelines for destination based on CTAS score, but there is allowance for triage nurse discretion in assignment of patient destination. A CTAS score of 2 (Emergent) is assigned to conditions that are a potential threat to life limb or function and it is recommended that patients be seen by a physician within 15 minutes 95% of the time [14,16]. We excluded patients if they were admitted to the hospital on the index visit. Patients were not excluded if they presented more than once. We divided the patients into cohorts by geographic destination in our department. Non-monitored patients were those who were assigned to the least acute area of our department. Monitored patients were those triaged to the most acute areas of our department and were placed on continuous cardiac and respiratory monitoring.

A single reviewer (EO) who was not blinded to the study hypothesis abstracted data onto a standardized Microsoft Excel spreadsheet. Data were obtained from the electronic health record, which contained scanned handwritten nursing triage notes and physician notes as well as computerized laboratory and imaging results. We recorded the following variables: triage time and date, triage score, triage destination, time of initial physician assessment, investigations ordered, and referrals made. We also recorded whether a patient’s destination changed during the ED visit. Up-triage was defined as a patient moved from the non-monitored area to the monitored area. Down-triage was defined as moving from the monitored area to the non-monitored area. Change in triage destination was made at the discretion of the bedside nurse or treating physician and could occur at any time during the patient’s stay in the ED. We do not have protocols in our ED for up-triage based on time to initial physician assessment.

### Study outcome measures

Study outcomes included: triage destination during ED crowding conditions, time to physician assessment (the time from patient arrival to assessment by a physician, in minutes), investigations ordered, and unscheduled return to our institution within 14 days of the index visit.

### ED crowding measure

ED crowding was measured using ED occupancy at the time of patient triage, which was defined as the ratio of total number of patients in the ED (admitted and not admitted) to the number of beds in the ED [17]. For our department, we included numbers of beds in all areas of the ED, monitored and non-monitored. ED occupancy is an accepted measure of ED crowding, but there is no universally accepted threshold that defines ED crowding [18,19]. We determined, based on local expert consensus, that an ED occupancy score of greater than 1.5 would indicate that our ED was crowded.

### Sample size and statistical analysis

A rate of return to our ED in this cohort of patients was assumed to be 20% (based on previous studies in our department) [20]. A difference in return rates of 10% was deemed significant, resulting in a sample size of 588. In this pilot study, the sample size of 500 charts was chosen for convenience. Descriptive statistics were used to report patient and system characteristics. Univariate analysis was performed with the χ^2^ test for all dichotomous variables. The Fisher exact test was used to analyze nominal variables and continuous variables were analyzed with the Wilcoxon two-sample test.

## Results

### Study flow

**Figure 1:**
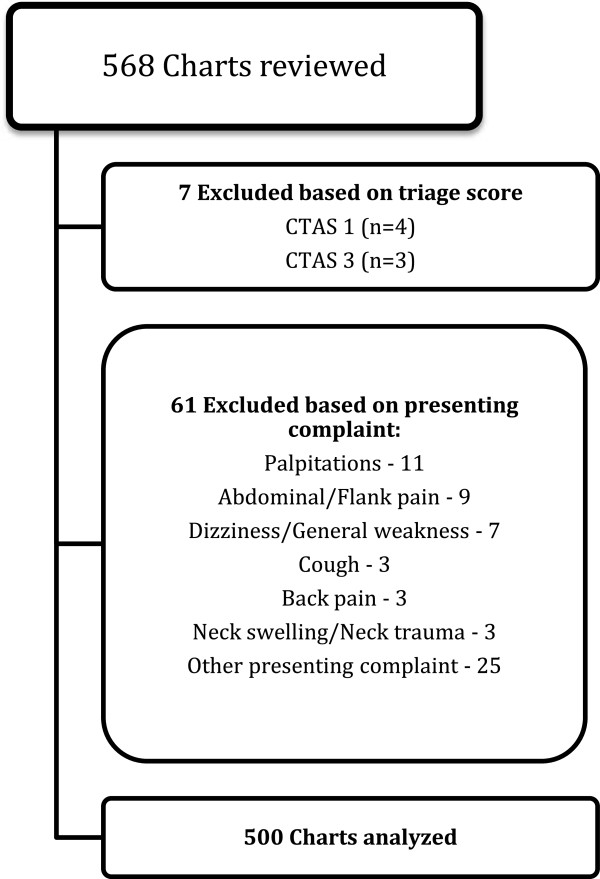
Study flow diagram.

We found 568 eligible health records during the study period; 7 visits were excluded for a CTAS score other than 2 and 61 charts were excluded based on chief complaint. Finally, we included a total of 500 health records in our study (Figure 1).

### Patient and system characteristics

Over half of the patients were male. Chest pain was the most common chief complaint. Approximately half of the included patients were triaged during crowded conditions. Most of the patients were triaged to the monitored area (Table 1).

**Table 1:** Patient and system characteristics for 500 high acuity patients presenting with chest pain or shortness of breath

**Patient characteristics**	**n (%)**
Male	269 (53.8)
Presenting complaint	
Chest pain	392 (78.4)
Shortness of breath	108 (21.6)
**System characteristics**	**n (%)**
ED crowding status^1^ at time of triage	
Crowded	260 (52.0)
Non-crowded	240 (48.0)
Triaged ED location	
Non-monitored	104 (20.8)
Monitored	396 (79.2)

^1^ED Occupancy = Number of patients in the ED/Number of beds in the ED. Crowded is defined as an ED Occupancy >1.5.

### Triage decision making and time to initial physician assessment

**Figure 2:**
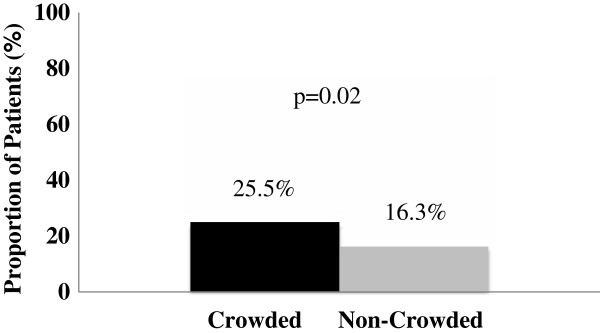
Proportion of patients triaged to the non-monitored area during crowded and non-crowded conditions (n = 500).

We found that patients who presented with a chief complaint of chest pain or shortness of breath and were assigned a CTAS score of 2 were triaged to the non-monitored area of the ED significantly more often when the ED was crowded (25.0% vs. 16.3%, *P* = 0.02) (Figure 2).

**Figure 3:**
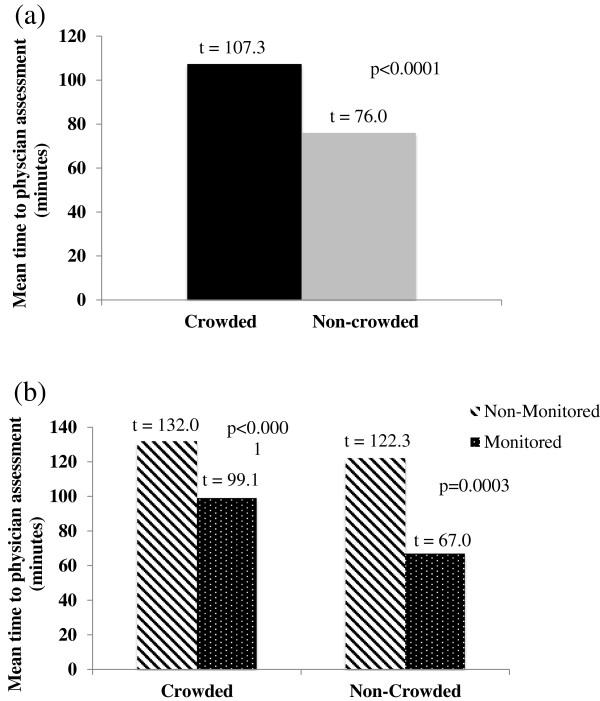
**Mean time from triage to physician initial assessment during crowded and non-crowded conditions (n = 500). (a)** Overall. **(b)** Divided by non-monitored and monitored areas.

The mean time to physician initial assessment was significantly longer for those patients triaged when the ED was crowded (107.3 minutes vs. 76.0 minutes, *P* <0.0001) (Figure 3a). The mean time to physician initial assessment was significantly longer in the non-monitored area of the ED than in the monitored area (Figure 3b). This was found to be true both when the ED was crowded (132.0 minutes vs. 99.1 minutes, *P* <0.0001) and when it was non-crowded (122.3 minutes vs. 67.0 minutes, *P* = 0.0003).

Patients were not up-triaged more often from the non-monitored area to a more acute area of the department when the ED was crowded than when the ED was non-crowded (3.1% (n = 8) vs. 1.3% (n = 3), *P* = 0.42).

### ED crowding and geographical influence on investigations ordered

**Figure 4:**
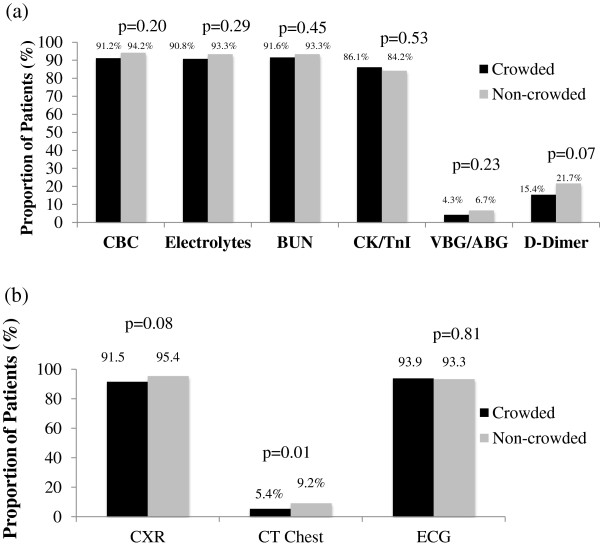
**Investigations ordered for high acuity ED patients triaged either to the non-monitored or monitored areas of the ED, presenting with chest pain or with shortness of breath. (a)** Blood-work ordered. **(b)** Imaging and ECGs ordered. [CBC, Complete Blood Count; BUN, Blood Urea Nitrogen; CK, Creatinine Kinase; TnI, Troponin I; VBG, Venous Blood Gas; ABG, Arterial Blood Gas; CXR, Chest Radiograph; CT Chest, Chest Computed Tomography; ECG, Electrocardiogram].

ED crowding did not appear to influence the proportion of patients who received ED investigations with the exception of more chest computed tomography ordered when the ED was not crowded (9.2% vs. 5.4%, *P* = 0.01) (Figure 4).

Data not depicted in the figures shows that investigations were influenced by the patient’s geographic location in the ED, with more investigations ordered in the monitored area of the ED: complete blood count: 99.2% vs. 67.31%, *P* <0.0001; electrolytes: 99.4% vs. 64.4%, *P* <0.0001; blood urea nitrogen: 99.2% vs. 66.4%, *P* <0.0001; venous or arterial blood gas: 6.6% vs. 1.0%, *P* = 0.03). Exceptions to this were significantly fewer electrocardiograms (86.5% vs. 95.5%, *P* = 0.0009) and D-dimers (26.0% vs. 16.4%, *P* = 0.03) ordered in the monitored area. We found no significant difference in the number of chest radiographs (*P* = 0.61) and chest computed tomography scans (*P* = 0.52) ordered. More patients received a referral to another service, either as an inpatient or as an outpatient from the monitored area (51/396 (12.8%) vs. non-monitored 1/104 (1%) *P* <0.0001).

### Proportion of return visits to the ED

**Figure 5:**
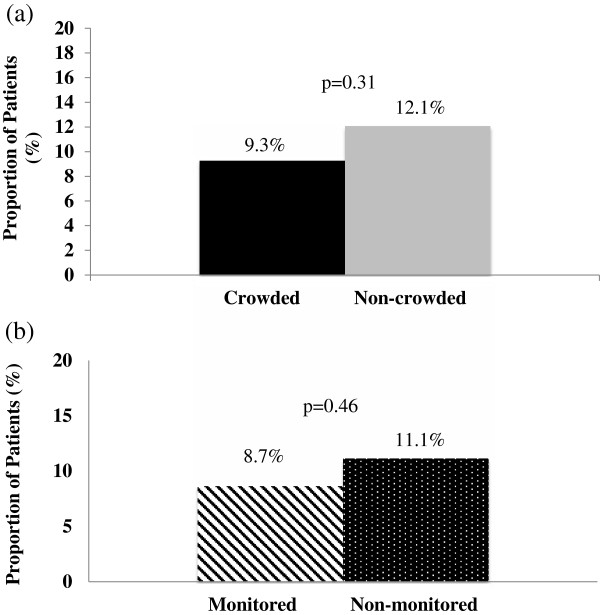
**Proportion of patients who return to the ED within 14 days of index visit. (a)** Patients triaged during crowded or non-crowded conditions. **(b)** Returns to the ED by initial triage destination.

Overall, rates of unscheduled returns to our ED were lower than anticipated: 51/500 (10.2%). We did not find a significant association between ED crowding and the proportion of patients who had an unscheduled return to the ED within 14 days of the index visit (9.3% vs. 12.1%, *P* = 0.31) (Figure 5a). In addition, the difference in geographic ED location (non-monitored vs. monitored) was not significantly associated with an increase in the proportion of patients with an unscheduled return to the ED (11.1% vs. 8.7%, *P* = 0.46) (Figure 5b).

## Discussion

Understanding how ED crowding affects patient outcomes has been identified as a research priority in emergency medicine [21]. Assignment of a triage score and destination in the emergency department has an important impact on the course of a patient’s ED visit [4]. We postulated that the negative impact of ED crowding with regard to increased patient mortality, increased adverse events, and delayed antibiotic administration in patients with pneumonia reported in previous studies may be related to differences in triage practices during times of crowding [5-10]. We hypothesized that patients would be triaged more often to the non-monitored or less acute area of our ED when the ED was crowded. We theorized that the patients in the non-monitored area would have fewer investigations and the physicians’ perception of the severity of their illness would be negatively impacted [4]. We found that high acuity patients were triaged to the non-monitored area of the ED more often when they presented during crowded conditions. This change in triage destination during crowded conditions may be based on an assumption by the triage nursing staff that patients would be seen more quickly by a physician if they are not required to wait for a monitored bed. This group of patients also received less blood work and fewer imaging tests than those sent to the monitored or more acute area of our ED. The differences in investigations and imaging tests ordered for these patients is potentially based on the physician’s perception of lower acuity of presentation because the patient is in the non-monitored area of the ED. A few of the differences were unexpected, in particular the fact that fewer ECGs were ordered in the monitored area of the ED. It is possible that because the patients were on continuous cardiac monitors that 12 lead ECGs were not obtained. Another possible explanation for this is that ECGs performed on these patients were lost and not scanned in to the computer record from which we obtained our data. We speculate that more D-dimers were ordered in the non-monitored area of the ED because it is used more as a “rule-out” test. For patients perceived to be more ill, a CT scan may have been ordered to investigate pulmonary embolism with a D-dimer omitted from the work-up. Overall, ED crowding led to more high-acuity patients being triaged to the non-monitored area of our ED, with fewer investigations performed.

We did not find that ED crowding conditions resulted in an increase in return visits to the ED within 14 days for patients presenting with chest pain or shortness of breath and assigned a CTAS score of 2. Given that we experienced overall rates of return that were lower than anticipated, our study may be underpowered to detect a true difference. This finding is consistent, however, with previous research by Hu et al., which found no correlation between unscheduled return visits to the ED and ED crowding [22]. We did show an increase in time to physician initial assessment for patients triaged during crowding conditions. A previous study by Guttman et al. showed a positive correlation between increased waiting times and admission to hospital or death within 7 days for discharged patients [23].

We used ED occupancy rate as our measure of ED crowding because it is a parsimonious, valid measurement of ED crowding. ED occupancy rate has been shown to perform as well as the following markers of ED crowding: EDWIN score, patients left without being seen and ambulance diversion times [16]. ED occupancy has the advantage of being calculable for the time of patient triage rather than as an average over the course of a day and hence may offer increased precision. In this pilot study, we attempted to specifically examine the effect of ED crowding on triage destination; therefore, a crowding measure specific for the time of triage was felt to be the most useful. In future work in this area we would measure crowding using the EDWIN score, ambulance diversion rates, and rates of left without being seen as well as using ED occupancy.

There are several limitations to this study. Firstly, it was restricted to a single institution that encompasses two large tertiary care EDs and we were only able to detect return visits to our institution and not to other institutions in our area. Previous studies conducted at our center indicate that patients rarely return to other institutions after an index visit to our ED [20]. Our sample size was relatively small, with only 500 health records included. Previous studies at our institution estimated a rate of return in this cohort of 20% [20]. However, in the group of patients we studied our rate of return was 10.2%, meaning that our study was under-powered. We set a cut-off for ED crowding at an occupancy level of 1.5 – this may be too low to capture the times when our department was under the most stress. The cut-off was based on local expert consensus rather than literature-based, as there is no accepted threshold for ED crowding using ED occupancy in the literature. It is possible that a higher ED occupancy level would show a larger difference between the groups. Also, ED occupancy was calculated at the time of triage, reflecting only occupancy at that one time. Change in occupancy level throughout the patients’ stay in the ED may also have affected the investigations ordered and potentially the rate of return to the ED. Due to the retrospective nature of the study design, we were unable to determine if CTAS scores were appropriately assigned for the patients in the study. Previous literature suggests that assignment of CTAS score has a good inter-rater reliability [24,25]. However, it is possible that ED crowding influenced assignment of CTAS score. This study was based on the assumption that patients with the same chief complaint and the same CTAS score have the same level of acuity and theoretically then need the same amount of monitoring and investigation, which may not be true.

This is the only study so far to examine the impact of ED crowding on triage destination. A larger, multi-center study would be necessary to determine whether changes in triage destination have a negative impact on patient outcomes. As triage is the first point of contact with ED staff, this interaction influences the patient’s entire stay in the department and should be an important area for future ED crowding research.

## Conclusions

In this pilot study, we found that during crowded conditions, high acuity patients presenting with chest pain or shortness of breath had a higher rate of triage to the non-monitored area of the ED, longer times to physician initial assessment, and an associated lower rate of investigations. Despite these findings, with our small sample size we could not detect a difference in rates of unscheduled returns to our institution. Future research should be directed at examining whether these changes in triage destination during crowded conditions lead to worse patient outcomes.

## Abbreviations

CTAS: Canadian Triage and Acuity Scale; ED: Emergency Department.

## Competing interests

The authors declare that they have no competing interests.

## Authors’ contributions

EO conceived of the study, gathered data, and drafted the manuscript. MG revised the manuscript. CW gathered data and revised the manuscript. LC supervised development of the study design and coordination and revised the manuscript. All authors read and approved the final manuscript.
